# Neurological abnormalities in 97 dogs with detectable pituitary masses

**DOI:** 10.1080/01652176.2019.1622819

**Published:** 2019-05-21

**Authors:** Marika Menchetti, Luisa De Risio, Greta Galli, Giunio Bruto Cherubini, Daniele Corlazzoli, Massimo Baroni, Gualtiero Gandini

**Affiliations:** a Department of Veterinary Medical Sciences, University of Bologna, Ozzano dell'Emilia, Italy;; b Department of Veterinary Neurology, The Animal Health Trust, Newmarket, Suffolk, UK;; c Dick White Referrals, Six Mile Bottom, Cambridgeshire, UK;; d Policlinico Veterinario Roma Sud, Rome, Italy;; e Clinica Veterinaria Valdinievole, Monsummano Terme, Italy

**Keywords:** Dog, canine, pituitary, mass, neurology

## Abstract

**Background:** Pituitary tumours are common neoplasms of the sellar region in small animals. However, detailed information regarding the spectrum and severity of possible neurological signs are lacking.

**Objective:** To retrospectively describe the neurological abnormalities in a population of dogs with a detectable pituitary mass (DPM) and relate them with the size of the mass and magnetic resonance imaging (MRI) signs of brain compression (BC). Client-owned dogs were included in the study if they had MRI showing a DPM and a detailed neurological examination. The neurological signs were evaluated in relation to the pituitary height/brain ratio (P:B ratio) and the presence/absence of brain compression.

**Results:** Ninety-seven dogs were enrolled. Besides abnormal mentation and behaviour (77%), gait (61%) and cranial nerve abnormalities (44%), other unreported neurological signs observed included postural abnormalities (21%), pain and/or hyperesthesia (25%) and abnormal postural and proprioceptive reactions (49%). The majority of dogs with DPM had signs of BC. The presence of a high pituitary height/brain area and BC represented a risk factor for developing mental status abnormalities.

**Conclusion:** Neurological signs recorded in DPM-affected dogs include not only the typical forebrain signs but also gait disturbances and hyperesthesia. Neurological signs are positively associated with increased P:B ratio and MRI signs of brain compression.

## 1. Introduction

In human and veterinary literature, it is well known that pituitary tumours (PTs) can produce neurological dysfunction. In human medicine, PTs can be classified as a ‘microtumor’ when the mass has a diameter <10 mm, and as a ‘macrotumor’ when the mass has a diameter ≥10 mm (Ihle 1997; Moore and O’Brien 2008; Trouillas et al. 2013).

Pituitary macrotumours (PMaTs) are considered more likely to be associated with the development of neurological signs than pituitary microtumours (PMiT) (Nelson et al. 1989; Katznelson et al. 1998; Thèon and Feldman 1998; Moore and O’Brien 2008). It has been estimated that 10 to 30% of dogs with pituitary-dependent hyperadrenocorticism (PDH) will eventually develop a PT causing neurological signs (Bertoy et al. 1996). In the dog, the effectiveness of classification of micro- and macrotumours was extensively debated because pituitary adenomas that are between 6 and 10 mm in height enlarge the gland and therefore cannot be classified as microtumours (Nelson et al. 1989; Meij et al. 2002; Moore and O’Brien 2008).

To overcome this controversy, Kooistra et al. (1997) defined ‘detectable pituitary masses’ (DPM) when the dorsal margin of the pituitary gland protruded above the suprasellar extensions of the intercrural cistern. The same authors proposed the ‘pituitary height to brain area ratio’ (P:B ratio) to objectively evaluate whether the pituitary is enlarged or not, in relation to the brain. According to their results, the P:B ratio had the highest discriminatory power in distinguishing enlarged from non-enlarged pituitaries (Kooistra et al. 1997). The P:B ratio is obtained using both a CT and a post-contrast T1W transverse image of the pituitary gland, measuring the height of the pituitary gland and tracing the edges of the brain, and finally calculating the ratio of the pituitary gland height (mm) to the brain area (mm^2^) x100 (Kooistra et al. 1997; Auriemma et al. 2009).

In the dog, although it is well recognized that PTs may cause neurological dysfunction by compressing and/or invading the adjacent central nervous system (CNS) structures, not all affected patients show neurological abnormalities (Ihle 1997; Moore and O’Brien 2008). Detailed data regarding the neurological presentation and the results of the neurological examination of dogs with PMaTs are almost lacking. In textbooks, the most common signs in dogs with a pituitary macroadenoma include altered mentation, compulsive gait and pacing, while ataxia, blindness and seizures are less commonly reported (Behrend 2015). Neurologic signs are described in general terms in a large case series focused on the diagnostic findings in dogs affected by PMaT with, or without neurologic abnormalities (Wood et al. 2007). Hence, the aims of this retrospective study were to provide a detailed description of the neurological findings in a population of dogs with a detectable pituitary mass. Furthermore, neurological findings were related to the size of the mass, calculated using the P:B ratio, and MRI signs compatible with brain compression.

## 2. Materials and methods

A multicentre retrospective study was performed, reviewing medical records of dogs referred to the neurology/neurosurgery services of five veterinary referral hospitals between January 2000 and December 2015.

Dogs were included in the study if they had a medical record comprising a detailed neurological examination and a MRI study showing a DPM. MRI findings were considered consistent with DPM if the dorsal margin of the pituitary gland protruded above the suprasellar extensions of the intercrural cistern, as previously described (Kooistra et al. 1997).

In order to minimise uncertainty on the origin of the neurological signs, dogs with history of treatment with mitotane and dogs showing previous recurrence of seizures were excluded from the study. Similarly, based on the data in the clinical records, all dogs with concurrent disorders potentially producing neurological signs were excluded.

In absence of histologic confirmation, only midline masses arising from the sellar region were included. MRI findings possibly related to other types of neoplasia (i.e. lateralization, dural tail sign, masses whose origin was not originating from the sellar region, multiple masses) were evaluated and led to the exclusion of further cases. For the above mentioned reasons, the term ‘DPMs’, in line with previous report, was used to define the space-occupying lesions of the dogs included in the study (Kent et al. 2007).

A Diplomate of the European College of Veterinary Neurology (ECVN) or a neurology resident performed all the neurological examinations. All MRIs were interpreted and reported by a Diplomate of the European College of Veterinary Diagnostic Imaging (ECVDI) or ECVN Diplomate at the time of referral. For the purpose of this study, all MRI studies were reviewed by an ECVN Diplomate or an experienced ECVN resident blinded to the neurological examination findings.

The study was approved by the Animal Health Trust Clinical Research Ethics Committee, Animal Health Trust, New Market (AHT 10-2015).

### 2.1. Neurological signs

Neurological signs were classified as abnormalities in mental status and behaviour, posture, gait, proprioceptive and postural reactions and cranial nerves. Epileptic seizure occurrence was also recorded. Due to the retrospective nature of the study, data regarding the type and detailed frequency of seizures were not always available. Data regarding pain or hyperesthesia were described with specific attention to the localization including the head, the cervical, thoracic or lumbar spine. Four intervals were used to describe the duration of the neurological signs before the referral: less than 1 month, between 1 and 3 months, between 3 and 6 months and more than 6 months. Survival time was defined as the time from the date of MRI to the date of euthanasia.

### 2.2. Diagnostic imaging

MRI of the brain was performed using either high-field or low-field equipment and, specifically, 1.5 Tesla (T) (Signa Echo Speed MRI scanner, GE healthcare, Chicago, USA); 0.4 T (Aperto permanent magnet, Hitachi, Tokyo, Japan); 0.3 T (AIRIS 2, Hitachi, Tokyo, Japan); 0.2 T (Vet-MR, ESAOTE, Genova, Italy) and 0.22 T (MrJ, Paramed, Genova, Italy). Dogs were scanned under general anaesthesia in sternal or dorsal recumbency according to the different coils and protocols. The images evaluated were obtained by using transverse and sagittal pre and post-contrast T1-weighted (T1W) and T2-weighted (T2W). Different commercially available paramagnetic contrast mediums were used at a dose of 0.1 mmol/kg BW IV and included gadodiamide, gadobenate dimeglumine, gadobutrol and gadoteric acid. The presence of brain compression (BC), defined as displacement of regional brain structures and/or the presence of hyperintensity in T2W and FLAIR images attributable to parenchymal oedema, was recorded and used for statistical purposes to distinguish two groups based on the presence or absence of BC.

### 2.3. P:B ratio

**Figure 1.**
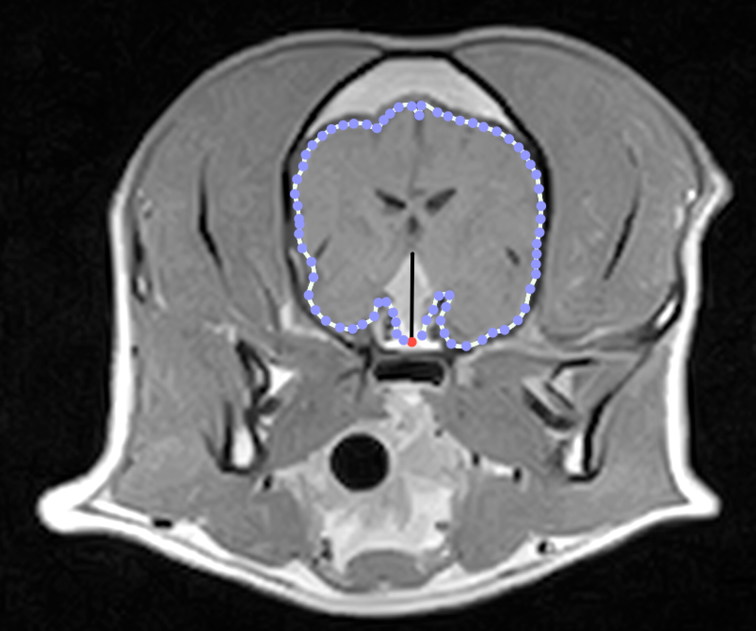
Transverse post-contrast T1-W MR image of the brain from a dog with a DPM. The P:B ratio was calculated as the ratio of the pituitary gland height (vertical line) to the brain area (dotted line) x 100.

In each dog, the P:B ratio was calculated using transverse sections from gadolinium-enhanced T1W MR images in which the PT was at its major extension in the cranial-caudal and dorsal directions, as previously described (Kooistra et al. 1997). In particular, the height of the pituitary mass was measured in the post-contrast transverse T1W image showing its largest cross-section. In the same section, the edges of the brain were traced, and the enclosed area was obtained by the computer software (OsiriX Medical Imaging Software). Hence, the P:B ratio was calculated as the ratio of the pituitary gland height (mm) to the brain area (mm^2^) x 100 (Figure 1) (Kooistra et al. 1997; Auriemma et al. 2009). The pituitary gland was considered enlarged (Enlarged Detectable Pituitary Mass; En-DPM) if the P:B ratio was > 0.31(mm^−1^) and non-enlarged (non-Enlarged Detectable Pituitary Mass; nEn-DPM) if the P:B ratio was ≤0.31(mm^−1^) (Kooistra et al. 1997; Auriemma et al. 2009).

### 2.4. Statistical analysis

Data analysis was performed using a statistical analysis software (PAST 3.x, Hammer & Harper, University of Oslo, Norway), while calculations and graphs were obtained using an electronic spreadsheet (Microsoft Excel, Redmond, Washington, USA).

For statistical purposes, the study population was divided in two groups based on the P:B ratio results: dogs with enlarged DPM (En-DPM) and dogs with non-enlarged DPM (nEn-DPM). Additionally, the study population was divided in two other groups based on presence or absence of BC (respectively: BC; no-BC). Descriptive analysis was made on the general population and on the four different groups above mentioned. The neurological signs were correlated with the DPM size and with the presence/absence of BC. For the analysis of the duration of the neurological signs, a cut-off of 1 month was used to perform the statistics. The associations between categorical variables were assessed using the chi-squared test or Fisher’s exact test depending on whether the value in one or more of the cells of the contingency table was five or less. Odds ratios (OR) and 95% confidence intervals (CIs) were calculated for variables, when those did not contain 0 as value. P values were considered significant when <0.05 and when the 95% CI of the OR excluded 1.0.

## 3. Results

### 3.1. Signalment data

Ninety-seven dogs met the inclusion criteria. 84% (81/97) were purebred dogs, with the Boxer (20%; 16/81) and Labrador Retriever (14%; 11/81) being most highly represented, while mixed breed dogs accounted for 16% of the population (16/97). The median age at the time of the neurological examination was 9 years (range 3–14 years). The study group comprised 45 females (46%; 37 spayed: 38%) and 52 males (54%; 29 neutered: 30%). The median body weight was 23.2 kg (range 3–76 kg). Duration of the clinical signs prior to referral was less than one month in 55 dogs (57%), between 1 and 3 months in 22 (23%), between 3 and 6 months in 12 (12%) and more than 6 months in 8 dogs (8%).

### 3.2. Data on treatment, assessment of hyperadrenocorticism (HAC), and presence of concurrent endocrinopathies

Data regarding treatment were retrospectively available for 59 dogs (61%). Of those, 35 dogs received medical treatments (59%), 15 dogs had both medical and radiotherapy treatments (25%), 4 dogs underwent radiotherapy alone (7%) and 5 dogs were surgically treated (9%).

57 dogs (59%) were tested for HAC and 36 dogs (63%) had a final diagnosis of HAC. Of these, 31 had an En-DPM (86%) and 5 had a nEn-DPM (14%). All dogs with HAC were treated with trilostane, including two dogs that also had radiotherapy (supplementary material Table 1: Information from available patient records concerning treatment, assessment of HAC and concurrent endocrinopathies).

**Table 1.** Significant results of univariate analysis to identify factors associated with enlarged or non-enlarged DPMs.

Factors	No. (%) En-DPM	No. (%) nEn-DPM	Chi^2^*P*-value	OR	95% CI	*P*-value
Presence or not of brain compression						
BC	62 (73%)	2 (17%)	< 0.001*	13.47	2.74–66.22	< 0.001*
no-BC	23 (27%)	10 (83%)				
Duration of clinical signs before referral						
≤1 month	43 (78%)	12 (22%)	< 0.01*	\	\	\
>1 month	42 (100%)	0 (0%)				
Mental status and behaviour						
Altered	71 (92%)	6 (8%)	0.01*	5.07	1.43–18.03	0.01*
Normal	14 (70%)	6 (30%)				
Obtundation						
Present	54 (95%)	3 (5%)	0.01*	5.23	1.32–20.76	0.02*
Absent	31 (77%)	9 (23%)				
Disorientation						
Present	24 (100%)	0 (0%)	0.03*	\	\	\
Absent	61 (84%)	12 (16%)				
Posture						
Altered	20 (100%)	0 (0%)	0.05	\	\	\
Normal	65 (84%)	12 (16%)				
Circling						
Present	22 (100%)	0 (0%)	0.045*	\	\	\
Absent	63 (84%)	12 (16%)				
Epileptic seizures						
Yes	12 (71%)	5 (29%)	0.02*	0.23	0.06–0.84	0.01
No	73 (91%)	7 (9%)				

\= not applicable; * = significant *P*-values; OR = odds ratio.

Seventeen dogs (18%) showed concomitant endocrinopathies. Of those, 14 had hypothyroidism and 3 had diabetes insipidus (supplementary material Table 1: Information recorded on available clinical records concerning treatment, assessment of HAC and concurrent endocrinopathies).

### 3.3. Neurological signs

All the dogs showed at least one neurological sign at the time of referral. The neurological abnormalities detected are detailed in Supplementary 2 (supplementary material Table 2: Details of the neurological abnormalities detected on the overall population).

**Table 2.** Significant results of univariate analysis to identify factors associated with brain compression in the case of DPMs.

Factors	No. (%) BC	No. (%) no – BC	Chi^2^*P*-value	OR	95% CI	*P*-value
Duration of clinical signs before referral						
≤1 month	29 (53%)	26 (47%)	< 0.01*	0.22	0.08–0.59	< 0.01
>1 month	35 (83%)	7 (11%)				
Mental status and behaviour						
Altered	55 (71%)	22 (29%)	0.03*	3.06	1.11–8.39	0.03*
Normal	9 (45%)	11 (55%)				
Disorientation						
Present	22 (92%)	2 (8%)	< 0.01*	8.12	1.78–37.13	< 0.01*
Absent	42 (58%)	31 (42%)				
Emprosthotonus						
Present	7 (100%)	0 (0%)	0.049*	\	\	\
Absent	57 (63%)	33 (37%)				
Proprioceptive and postural reactions						
Altered	36 (77%)	11 (23%)	0.03*	2.57	1.07–6.18	0.03*
Normal	28 (56%)	22 (44%)				
Menace response						
Altered	23 (85%)	4 (15%)	0.01*	4.07	1.27–13.02	0.02*
Normal	41 (59%)	29 (41%)				
Pain						
Present	20 (83%)	4 (17%)	0.045*	3.3	1.02–10.63	0.046*
Absent	44 (60%)	29 (40%)				

\= not applicable; * = significant *P*-values; OR = odds ratio.

Mental status (consciousness and behaviour) was abnormal in 77 dogs (79%). The most frequent deficit described was obtundation (*n* = 45; 58%). Compulsive behaviour and disorientation were observed in 21% (*n* = 16) and 16% (*n* = 12) of the dogs, respectively. Posture was abnormal in 20 dogs (21%). The most frequently observed abnormal posture was emprosthotonus (low head carriage; *n* = 7; 35%). All seven dogs with emprosthotonus had mental status abnormalities and five out of them showed concurrent cervical and/or head hyperalgesia.

Fifty-nine dogs (61%) showed abnormal gait, consisting mostly in four limbs ataxia (*n* = 42; 71%) and circling (*n* = 22; 37%).

Proprioceptive and postural reaction deficits were present in 47 dogs (49%). Cranial nerve deficits were observed in 43 dogs (44%), including abnormal menace response (*n* = 27; 63%), change in pupillary size (*n* = 12; 28%) and pupillary light reflex (PLR) (*n* = 14; 33%).

The patients’ owners observed epileptic seizures in 17 dogs (18%).

Pain and/or hyperesthesia were elicited on palpation in 24 dogs (25%) with major prevalence of cervical region (*n* = 12; 50%), head region (*n* = 5; 21%) and head and cervical region (*n* = 4; 17%).

### 3.4. Survival time

Data regarding the survival time were available in 44 dogs (45%). 24 (55%) were euthanized at the time of diagnosis. The median survival time of the remaining 20 dogs was 75 days (range 30-1080 days). Causes of death/euthanasia were retrospectively available only for 3/20 dogs and consisted of a gastric dilation and volvulus, deterioration of neurological signs and deterioration of HAC. Survival time was not related to the P:B ratio (*P* = 0.6) or the presence of brain compression (*P* = 0.4).

### 3.5. Clinical variables associated with En- and nEn-DPMs

P:B ratio assessment showed an En-DPM in eighty-five dogs (88%). Out of these, 62 dogs (73%) showed signs of BC (*P* < 0.001) (Table 1).

All dogs with neurological abnormalities enduring for more than 1 month had an En-DPMs (*P* < 0.01) and were more likely to have mental status abnormalities than dogs with a nEn-DPM (*P* = 0.01) (Table 1).

Detailed results of the univariate analysis to identify factors associated with Enlarged or non-Enlarged DPMs can be found in supplementary material Table 3.

### 3.6. Clinical variables associated with BC

MRI signs of BC were detected in 64 dogs (66%). Dogs with altered mental status had significantly higher odds to have BC than other dogs (*P* = 0.03) (Table 2).

A significant association was observed also between postural and proprioceptive reaction abnormalities and BC (*P* = 0.03) (Table 2).

Menace response abnormalities were detected in 85% (23/27) of dogs with BC, representing a significant risk factor for BC (*P* = 0.02).

Dogs showing pain at the neurological examination were 3.3 times more likely to have BC (*P* < 0.045) (Table 2). Out of the population of dogs showing pain and BC (20/24; 83%), 55% (11/20) had pain localized at the cervical region. Detailed results of univariate analysis to identify factors associated with the presence of brain compression can be found in supplementary material Table 4.

## 4. Discussion

This study provides a detailed description of neurological signs in dogs with DPMs and investigates the relationship between DPM size and intracranial compression using the P:B ratio.

PTs are the most common tumours of the region of the sella turcica (Nelson et al. 1989; Wisner et al. 2011). Some PTs tend to grow dorsally, compressing the overlying diencephalon and causing, as a consequence, neurological signs (Nelson et al. 1989; Pollard et al. 2010). There is still controversy if the neurological signs observed in these dogs are related to the size of the mass alone or if they are a consequence of the growing pattern (Wood et al. 2007).

To date, the majority of data regarding neurological signs related to DPMs are found in retrospective studies of dogs with PDH (Nelson et al. 1989; Kipperman et al. 1992; Duesberg et al. 1995; Thèon and Feldman 1998; Wood et al. 2007; Pollard et al. 2010). Unfortunately, in most of these studies complete and detailed information concerning all neurological examinations were not available. In the largest case series describing neurological signs in 157 dogs with PDH, due to the limited information available on some patients, the authors decided to categorize the tumour size with respect to 'non-specific neurological signs' versus CNS-specific signs (Wood et al. 2007).

Our study documented multiple abnormalities detected on the neurological examination. Specifically, significant mental status, behaviour, posture, gait, postural reactions and cranial nerve abnormalities as well as the presence of pain and/or hyperesthesia were recorded. Noteworthy, postural abnormalities, pain and/or hyperesthesia and abnormal postural and proprioceptive reactions have never been reported in previous studies in dogs with DPMs.

Not surprisingly, a significant association was found between mental status and behaviour abnormalities and EN-DPM and BC. The detected mental status abnormalities were in agreement with previous studies, in which the majority of cases showed obtundation (Duesberg et al. 1995; Ihle 1997; Thèon and Feldman 1998; Wood et al. 2007). Our results support the hypothesis that, at least in presence of an En-DPM, mental status alterations have a neurological origin.

In our study, all dogs showing emprosthotonus had BC and the majority of them showed pain at the level of cervical region and/or head. It is well known that emprosthotonus may occur in association with intracranial disease, resulting in compression or stretching of the meninges or cerebral vasculature and referred pain (De Risio 2014; De Lahunta et al. 2015). In accordance with this hypothesis, our results showed that 25% of the population had pain and/or hyperesthesia on palpation at the level of the head and the cervical region, suggesting that pain at these locations may be considered referred pain related to the presence of BC. The retrospective nature of this study prevents the rigorous exclusion of concurrent etiologies and further studies, including MRI of the cervical/cervicothoracic regions of the spinal cord, are necessary to confirm this hypothesis.

Our findings on gait abnormalities are in agreement with previous studies, which described ataxia and circling as the most common gait alterations (Duesberg et al. 1995; Ihle 1997; Thèon and Feldman 1998; Wood et al. 2007). 90% of ataxic dogs had an En-DPM and 67% had signs of BC on MRI. Regrettably, the type of ataxia was not described in all clinical records, preventing appropriate reasoning about the possible pathophysiological mechanisms. The presence of ataxia in a high percentage of dogs which are expected to have mainly forebrain signs is an interesting issue that should be clarified by further studies.

Postural and proprioceptive reaction deficits, recorded in 48% of dogs, were significantly associated to the presence of BC (*P* = 0.03). This data may be explained by the compression of the proprioceptive pathways in the diencephalon, above the pituitary gland by the growing DPM. Despite numerous reports describing dogs with PTs (Sarfaty et al. 1988; Nelson et al. 1989; Kipperman et al. 1992; Duesberg et al. 1995; Kent et al. 2007; Wood et al. 2007; Moore and O’Brien 2008), postural and proprioceptive reactions deficits have not been reported before. Possible reasons for this discrepancy include the lack of focus on the neurological examination in previous studies.

The most represented deficit on the cranial nerves examination was the absent/decreased menace response. This finding is not surprising since PTs are expected to produce forebrain signs.

The pupillary abnormalities and PLR deficits recorded in a number of patients are possibly explained by the chiasmatic compression due to the presence of an enlarged sellar mass. In people, visual deficits are a common finding in cases of PTs, as the *diaphragma sellae* perfectly covers the pituitary fossa, allowing tumour growth toward the optic chiasm (Rhoton 2001; Alleyne et al. 2002; Ferrante et al. 2006; Ogra et al. 2014). In contrast, the pituitary fossa of the dog is covered by an incomplete *diaphragma sellae*, facilitating the dorsal growth of the neoplasm and is less likely to involve of the optic chiasm (Meij 2001; Hullinger 2013). Our results are in accordance with what is reported in the literature, where the visual deficits are represented in a minor percentage of dogs with DPMs (Sarfaty et al. 1988; Kipperman et al. 1992; Duesberg et al. 1995; Ihle 1997; Thèon and Feldman 1998; Wood et al. 2007; Moore and O’Brien 2008).

In the present study, the vast majority of the dogs with an En-DPM had MRI signs of BC. This finding may be easily explained as the direct consequence of the compressive effect of a growing mass on the brain. Previous studies have already reported either the presence of BC in dogs with PDH and neurological signs (Wood et al. 2007) and the association between severity of neurological signs and the PT volume (Nelson et al. 1989; Duesberg et al. 1995).

Unfortunately, our study does not contribute to the debate aimed to clarify whether the neurological signs are related to the size of the mass alone or depend also on the pattern of growth (Wood et al. 2007).

Due to the retrospective nature, the study has some limitations. The main study limitation is the lack of histopathologic evaluation. The authors cannot exclude that non-secreting tumours could be neoplasms of different origin, such as meningiomas. However, it is well known that in the vast majority of cases, the presence of a mass in the pituitary fossa on MRI is consistent with a neoplasm originating from the pituitary gland, the most common tumour of the region of the *sella turcica* (Wisner et al. 2011; Rissi 2015).

The presence of at least one neurological sign in all dogs could be the result of the inclusion criteria, aimed to emphasize the presence of a complete neurological examination. Authors consider this potential bias a minor weakness, since the main aim of the report was to describe in details the neurological signs in a population of dogs with DPM. Nevertheless, the dogs included in the study were referred both because of neurological signs and by colleagues of the internal medicine service who requested a neurological consultation to exclude subtle neurological abnormalities potentially unnoticed by the owners.

Involving different veterinary hospitals, both high- and low-field MRI studies were evaluated. The main limitation in evaluating low-field MRI sequences could be some inaccuracy in measuring the P:B ratio. However, low-field MRI has been shown to be a reliable method to measure P:B ratio by Auriemma et al. (2009). For the abovementioned reason, different MRI contrast agents were used. Contrast media solutions may have different pharmacokinetic and magnetic properties, potentially influencing the MRI findings (Rohrer et al. 2005). All the contrast agents used in this study were low molecular-mass gadolinium chelates and the effect of different pharmacokinetic profiles of contrast media on MRI findings are not documented in dogs with pituitary masses.

To avoid weaknesses linked to the retrospective nature of the study, our results should be confirmed by prospective studies planned using a specific and standardized diagnostic protocol, in order to exclude, with the greatest accuracy, other pathologies or medical treatments that could affect the mental status or cause neurological signs.

## 5. Conclusions

In conclusion, despite the abovementioned caution in the interpretation of the results, this study indicates that DPMs are associated with a variety of neurological abnormalities, including both forebrain signs as well as gait disturbances and hyperesthesia. The most frequent observed signs were abnormal mentation and behaviour, gait and postural reactions. Cervical hyperesthesia, recorded in dogs with DPMs and signs of BC, may reflect referred pain due to the intracranial mass.

Neurological signs were positively associated with increased P:B ratio and MRI signs of brain compression, indirectly suggesting a possible relationship with the size of the DPM.

Our results confirmed that, in case of clinical suspicion of a DPM, the neurological examination should be always thoroughly performed. Detection of neurological abnormalities should be adequately considered in order to proceed with a correct diagnostic work-up, which includes brain MRI, and plan a proper treatment.

## Supplementary Material

Supplemental Material
