# Supplementary material for: Neurological abnormalities in 97 dogs with detectable pituitary masses
Source: Vet Q. 2019 May 21;39(1):57–64. doi: 10.1080/01652176.2019.1622819 (PMC6831018; doi:10.1080/01652176.2019.1622819)
Supplement: Supplemental Material [file TVEQ_A_1622819_SM3317.zip › Supplementary_Table_1.docx]

Supplementary Table 1: Information from available patient records concerning treatment (N=59 [61%]), assessment of hyperadrenocorticism (N=57 [59%]) and concurrent other endocrinopathies (N=17 [18%]).

| **Treatment** (N=59 [61%]) | Medical treatment only 35 (59%) *  - antiepileptic drugs 6/35  - gabapentin 1/35  - corticosteroids 14/35  - antibiotics 2/35  - trilostane 15/35 |
| --- | --- |
|  | Medical treatment + Radiotherapy 15 (25%) *  - corticosteroids 14/15  - trilostane 3/15  - antiepileptic drugs 2/15 |
|  | Radiotherapy 4 (7%) |
|  | Surgery 5 (9%) |
| **Hyperadrenocorticism assessment (HAC)** (N=57 [59%]) | Positive endocrine tests for HAC 36/57 (63%)  Negative endocrine tests for HAC 21/57 (37%)  **Test used:**  - ACTH stimulation test  36/36  - Low doses desametasone suppression test 10/36  - Abdominal ultrasound 13/36 |
| **Concurrent endocrinopathies** (N=17 [18%]) | - hypothyroidism 14/17 - diabetes insipidus 3/17 |

* patients were medicated with more than one drug.
