# Supplementary material for: Neurological abnormalities in 97 dogs with detectable pituitary masses
Source: Vet Q. 2019 May 21;39(1):57–64. doi: 10.1080/01652176.2019.1622819 (PMC6831018; doi:10.1080/01652176.2019.1622819)
Supplement: Supplemental Material [file TVEQ_A_1622819_SM3317.zip › Supplementary_Table_2.docx]

Supplementary Table 2: Details of the neurological abnormalities detected on the overall population (97 dogs).

|  | **Abnormal N (%)** |
| --- | --- |
|  |  |
| **Mental status (consciousness and behavior)** | 77 (79%) * |
|  |  |
| Obtundation | 45 (58%) |
| Compulsive behaviour | 16 (21%) |
| Disorientation | 12 (16%) |
| Obtundation and disorientation | 12 (16%) |
| Head pressing | 13 (17%) |
| Star gazing | 9 (12%) |
| Urinating or defecating in unusual places | 8 (10%) |
| Aggressiveness | 7 (9%) |
| Circadian rhythm alterations | 4 (5%) |
|  |  |
|  |  |
| **Posture** | 20 (21%) * |
|  |  |
| Emprosthotonus | 7 (35%) |
| Head tilt | 4 (20%) |
| Postural instability | 3 (15%) |
| Recumbency | 2 (10%) |
| Head turn | 2 (10%) |
| Pleurothotonus | 2 (10%) |
| Abnormal based stance | 2 (10%) |
| Opisthotonus | 1 (5%) |
| Involuntary movements | 1 (5%) |
|  |  |
|  |  |
| **Gait** | 59 (61%) * |
|  |  |
| Four limbs ataxia | 42 (71%) |
| Circling | 22 (37%) |
| Tetraparesis | 12 (20%) |
| Paraparesis | 4 (7%) |
| Drifting | 2 (3%) |
| Tetraplegia | 1 (2%) |
| Weakness | 1 (2%) |
|  |  |
|  |  |
| **Proprioceptive and postural reactions** | 47 (49%) |
|  |  |
| Four limbs | 25 (53%) |
| Two limbs | 13 (28%) |
| One limb | 9 (19%) |
|  |  |
|  |  |
| **Cranial nerves** | 43 (44%) * |
|  |  |
| Menace response | 27 (63%) |
|  |  |
| Bilaterally absent | 11 (41%) |
| Bilaterally decreased | 6 (22%) |
| Unilaterally absent | 3 (11%) |
| Unilaterally decreased | 7 (26%) |
|  |  |
| Pupillary size | 12 (28%) |
|  |  |
| Anisocoria | 5 (41.7%) |
| Mydriasis | 5 (41.7%) |
| Miosis | 2 (16.6%) |
|  |  |
| Pupillary light reflex | 14 (33%) |
|  |  |
| Bilaterally absent | 7 (50%) |
| Bilaterally reduced | 5 (36%) |
| Unilaterally reduced | 2 (14%) |
|  |  |
| Others cranial nerves abnormalities | 16 (37%) |
|  |  |
| Visual deficits/loss of vision | 4 (25%) |
| Decreased/absent palpebral reflex | 4 (25%) |
| Nystagmus | 6 (37.5%) |
| Strabismus | 5 (31.2%) |
| Face asymmetry | 2 (12.5%) |
| Gag reflex deficit | 2 (12.5%) |
|  |  |

* Some patients showed more than one abnormality.
