# Supplementary material for: Neurological abnormalities in 97 dogs with detectable pituitary masses
Source: Vet Q. 2019 May 21;39(1):57–64. doi: 10.1080/01652176.2019.1622819 (PMC6831018; doi:10.1080/01652176.2019.1622819)
Supplement: Supplemental Material [file TVEQ_A_1622819_SM3317.zip › Supplementary_Table_3.docx]

Supplementary Table 3: Results of univariate analysis to identify factors associated with Enlarged or non-Enlarged DPMs.

| **Factors** | | **No. (%)**  **En-DPM** | **No. (%)**  **nEn-DPM** | **Chi^2^**  **P value** | **OR** | | **95% CI** | | **P value** |
| --- | --- | --- | --- | --- | --- | --- | --- | --- | --- |
| Breed | |  |  |  |  | |  | |  |
| Purebred | | 69 (85%) | 12 (15%) | 0.1 | \ | | \ | | \ |
| Mixed | | 16 (100%) | 0 (0%) |  |  |  |  |  |  |
| Age | |  |  |  |  | |  | |  |
| > 8.9 | | 46 (87%) | 7 (13%) | 0.78 | 0.84 | | 0.25 - 2.87 | | 0.78 |
| ≤ 8.9 | | 39 (89%) | 5 (11%) |  |  |  |  |  |  |
| Sex | |  |  |  |  | |  | |  |
| M | | 46 (88%) | 6 (12%) | 0.79 | 1.18 | | 0.35 - 3.95 | | 0.79 |
| F | | 39 (87%) | 6 (13%) |  |  |  |  |  |  |
| Weight | |  |  |  |  | |  | |  |
| > 23 Kg | | 43 (88%) | 6 (12%) | 0.97 | 1.02 | | 0.31 - 3.43 | | 0.97 |
| ≤ 23 Kg | | 42 (87%) | 6 (13%) |  |  |  |  |  |  |
| Presence or not of brain compression | | | | | | | | | |
| BC | 62 (73%) | | 2 (17%) | < 0.001^*^ | | 13.47 | | 2.74 - 66.22 | < 0.001^*^ |
| no-BC | 23 (27%) | | 10 (83%) |  | |  | |  |  |
| Duration of clinical signs before referral | | | | | | | | | |
| ≤ 1 month | | 43 (78%) | 12 (22%) | < 0.01^*^ | \ | | \ | | \ |
| > 1 month | | 42 (100%) | 0 (0%) |  |  |  |  |  |  |
| Survival time | |  |  |  |  | |  | |  |
| > 235 days | | 4 (57%) | 3 (43%) | 0.59 | 0.59 | | 0.09 - 3.98 | | 0.59 |
| ≤ 235 days | | 9 (69%) | 4 (31%) |  |  |  |  |  |  |
| Mental status and behavior | | | | | | | | | |
| Altered | | 71 (92%) | 6 (8%) | 0.01^*^ | 5.07 | | 1.43 - 18.03 | | 0.01^*^ |
| Normal | | 14 (70%) | 6 (30%) |  |  |  |  |  |  |
| Obtundation | |  |  |  |  | |  | |  |
| Present | | 54 (95%) | 3 (5%) | 0.01^*^ | 5.23 | | 1.32 - 20.76 | | 0.02^*^ |
| Absent | | 31 (77%) | 9 (23%) |  |  |  |  |  |  |
| Disorientation | |  |  |  |  | |  | |  |
| Present | | 24 (100%) | 0 (0%) | 0.03^*^ | \ | | \ | | \ |
| Absent | | 61 (84%) | 12 (16%) |  |  |  |  |  |  |
| Compulsion | |  |  |  |  | |  | |  |
| Present | | 15 (94%) | 1 (6%) | 0.42 | 2.36 | | 0.28 - 19.67 | | 0.43 |
| Absent | | 70 (86%) | 11 (14%) |  |  |  |  |  |  |
| Posture | |  |  |  |  | |  | |  |
| Altered | | 20 (100%) | 0 (0%) | 0.06 | \ | | \ | | \ |
| Normal | | 65 (84%) | 12 (16%) |  |  |  |  |  |  |
| Emprosthotonus | |  |  |  |  | |  | |  |
| Present | | 7 (100%) | 0 (0%) | 0.3 | \ | | \ | | \ |
| Absent | | 78 (87%) | 12 (13%) |  |  |  |  |  |  |
| Head tilt | |  |  |  |  | |  | |  |
| Present | | 4 (100%) | 0 (0%) | 0.44 | \ | | \ | | \ |
| Absent | | 81 (87%) | 12 (13%) |  |  |  |  |  |  |
| Head turn and/or pleurothotonus | | | | | | | | | |
| Present | | 4 (100%) | 0 (0%) | 0.44 | \ | | \ | | \ |
| Absent | | 81 (87%) | 12 (13%) |  |  |  |  |  |  |
| Circling | |  |  |  |  | |  | |  |
| Present | | 22 (100%) | 0 (0%) | 0.045^*^ | \ | | \ | | \ |
| Absent | | 63 (84%) | 12 (16%) |  |  |  |  |  |  |
| Ataxia | |  |  |  |  | |  | |  |
| Present | | 38 (90%) | 4 (10%) | 0.46 | 1.62 | | 0.45 - 5.78 | | 0.46 |
| Absent | | 47 (85%) | 8 (15%) |  |  |  |  |  |  |
| Tetraparesis | |  |  |  |  | |  | |  |
| Present | | 11 (92%) | 1 (8%) | 0.65 | 1.64 | | 0.19-13.94 | | 0.65 |
| Absent | | 74 (87%) | 11 (13%) |  |  |  |  |  |  |
| Proprioceptive and postural reactions | | | | | | | | |  |
| Altered | | 44 (94%) | 3 (6%) | 0.08 | 3.22 | | 0.81-12.72 | | 0.1 |
| Normal | | 41 (82%) | 9 (18%) |  |  |  |  |  |  |
| Cranial nerves | |  |  |  |  | |  | |  |
| Altered | | 40 (93%) | 3 (7%) | 0.15 | 2.67 | | 0.67-10.54 | | 0.16 |
| Normal | | 45 (83%) | 9 (17%) |  |  |  |  |  |  |
| Menace response | | | | | | | | | |
| Altered | | 26 (96%) | 1 (4%) | 0.11 | 4.85 | | 0.59-39.53 | | 0.14 |
| Normal | | 59 (84%) | 11 (16%) |  |  |  |  |  |  |
| Pupillary diameter | | | | | | | | | |
| Altered | | 12 (100%) | 0 (0%) | 0.16 | \ | | \ | | \ |
| Normal | | 73 (86%) | 12 (14%) |  |  |  |  |  |  |
| PLR | |  |  |  |  | |  | |  |
| Altered | | 13 (93%) | 1 (7%) | 0.52 | 1.99 | | 0.23 -16.72 | | 0.53 |
| Normal | | 72 (87%) | 11 (13%) |  |  |  |  |  |  |
| Epileptic seizures | |  |  |  |  | |  | |  |
| Yes | | 12 (71%) | 5 (29%) | 0.02^*^ | 0.23 | | 0.06 - 0.84 | | 0.01 |
| No | | 73 (91%) | 7 (9%) |  |  |  |  |  |  |
| Pain | |  |  |  |  | |  | |  |
| Present | | 23 (96%) | 1 (4%) | 0.16 | 4.08 | | 0.5 - 33.4 | | 0.19 |
| Absent | | 62 (85%) | 11 (15%) |  |  | |  | |  |

\ = not applicable; ^*^ = significant P values.
