# Supplementary material for: Neurological abnormalities in 97 dogs with detectable pituitary masses
Source: Vet Q. 2019 May 21;39(1):57–64. doi: 10.1080/01652176.2019.1622819 (PMC6831018; doi:10.1080/01652176.2019.1622819)
Supplement: Supplemental Material [file TVEQ_A_1622819_SM3317.zip › Supplementary_Table_4.docx]

Supplementary Table 4: Results of univariate analysis to identify factors associated with brain compression in case of DPMs.

| **Factors** | **No. (%)**  **BC** | **No. (%)**  **absent BC** | **Chi^2^**  **P value** | **OR** | **95% CI** | **P value** |
| --- | --- | --- | --- | --- | --- | --- |
| Breed |  |  |  |  |  |  |
| Purebred | 54 (67%) | 27 (33%) | 0.75 | 1.2 | 0.39 - 3.65 | 0.75 |
| Mixed | 10 (62%) | 6 (38%) |  |  |  |  |
| Age |  |  |  |  |  |  |
| > 8.9 | 35 (66%) | 18 (34%) | 0.99 | 1.01 | 0.43 - 2.34 | 0.99 |
| ≤ 8.9 | 29 (66%) | 15 (34%) |  |  |  |  |
| Sex |  |  |  |  |  |  |
| M | 33 (63%) | 19 (37%) | 0.57 | 0.78 | 0.34 - 1.83 | 0.57 |
| F | 31 (69%) | 14 (31%) |  |  |  |  |
| Weight |  |  |  |  |  |  |
| > 23 Kg | 31 (63%) | 18 (37%) | 0.57 | 0.78 | 0.34 - 1.82 | 0.57 |
| ≤ 23 Kg | 33 (69%) | 15 (31%) |  |  |  |  |
| Duration of clinical signs before referral | | | | | | |
| ≤ 1 month | 29 (53%) | 26 (47%) | < 0.01^*^ | 0.22 | 0.08 - 0.59 | < 0.01 |
| > 1 month | 35 (83%) | 7 (11%) |  |  |  |  |
| Survival time |  |  |  |  |  |  |
| > 235 days | 4 (57%) | 3 (43%) | 0.25 | 3 | 0.45 -20.15 | 0.26 |
| ≤ 235 days | 4 (31%) | 9 (69%) |  |  |  |  |
| Mental status and behavior | | | | | | |
| Altered | 55 (71%) | 22 (29%) | 0.03^*^ | 3.06 | 1.11 - 8.39 | 0.03^*^ |
| Normal | 9 (45%) | 11 (55%) |  |  |  |  |
| Obtundation |  |  |  |  |  |  |
| Present | 40 (70%) | 17 (30%) | 0.3 | 1.57 | 0.67 - 3.67 | 0.3 |
| Absent | 24 (60%) | 16 (40%) |  |  |  |  |
| Disorientation |  |  |  |  |  |  |
| Present | 22 (92%) | 2 (8%) | < 0.01^*^ | 8.12 | 1.78 -37.13 | < 0.01^*^ |
| Absent | 42 (58%) | 31 (42%) |  |  |  |  |
| Compulsion |  |  |  |  |  |  |
| Present | 13 (81%) | 3 (19%) | 0.16 | 2.55 | 0.67 - 9.68 | 0.17 |
| Absent | 51 (63%) | 30 (37%) |  |  |  |  |
| Posture |  |  |  |  |  |  |
| Altered | 15 (76%) | 5 (24%) | 0.34 | 1.71 | 0.56 - 5.22 | 0.34 |
| Normal | 49 (63%) | 28 (37%) |  |  |  |  |
| Emprosthotonus |  |  |  |  |  |  |
| Present | 7 (100%) | 0 (0%) | 0.049^*^ | \ | \ | \ |
| Absent | 57 (63%) | 33 (37%) |  |  |  |  |
| Head tilt |  |  |  |  |  |  |
| Present | 2 (50%) | 2 (50%) | 0.49 | 0.5 | 0.07 - 3.72 | 0.5 |
| Absent | 62 (67%) | 31 (33%) |  |  |  |  |
| Head turn and/or pleurothotonus | | | | | | |
| Present | 4 (100%) | 0 (0%) | 0.14 | \ | \ | \ |
| Absent | 60 (65%) | 33 (35%) |  |  |  |  |
| Circling |  |  |  |  |  |  |
| Present | 18 (82%) | 4 (18%) | 0.07 | 2.84 | 0.87 - 9.22 | 0.08 |
| Absent | 46 (61%) | 29 (39%) |  |  |  |  |
| Ataxia |  |  |  |  |  |  |
| Present | 28 (67%) | 14 (33%) | 0.9 | 1.06 | 0.45 - 2.47 | 0.9 |
| Absent | 36 (65%) | 19 (35%) |  |  |  |  |
| Tetraparesis |  |  |  |  |  |  |
| Present | 9 (75%) | 3 (25%) | 0.48 | 1.6 | 0.41 - 6.51 | 0.48 |
| Absent | 55 (65%) | 30 (35%) |  |  |  |  |
| Proprioceptive and postural reactions | |  |  |  |  |  |
| Altered | 36 (77%) | 11 (23%) | 0.03^*^ | 2.57 | 1.07 - 6.18 | 0.03^*^ |
| Normal | 28 (56%) | 22 (44%) |  |  |  |  |
| Cranial nerves |  |  |  |  |  |  |
| Altered | 31 (72%) | 12 (28%) | 0.26 | 1.64 | 0.69 - 3.89 | 0.26 |
| Normal | 33 (61%) | 21 (39%) |  |  |  |  |
| Menace response | | | | | | |
| Altered | 23 (85%) | 4 (15%) | 0.01^*^ | 4.07 | 1.27 -13.02 | 0.02^*^ |
| Normal | 41 (59%) | 29 (41%) |  |  |  |  |
| Pupillary diameter | | | | | | |
| Altered | 7 (58%) | 5 (42%) | 0.55 | 0.69 | 0.20 - 2.36 | 0.55 |
| Normal | 57 (67%) | 28 (33%) |  |  |  |  |
| PLR |  |  |  |  |  |  |
| Altered | 12 (86%) | 2 (14%) | 0.09 | 3.58 | 0.75 -17.05 | 0.11 |
| Normal | 52 (63%) | 31 (37%) |  |  |  |  |
| Epileptic seizures |  |  |  |  |  |  |
| Yes | 8 (47%) | 9 (53%) | 0.07 | 0.38 | 0.13 - 1.11 | 0.08 |
| No | 56 (70%) | 24 (30%) |  |  |  |  |
| Pain |  |  |  |  |  |  |
| Present | 20 (83%) | 4 (17%) | 0.045^*^ | 3.3 | 1.02 -10.63 | 0.046^*^ |
| Absent | 44 (60%) | 29 (40%) |  |  |  |  |

\ = not applicable; ^*^ = significant P values.
